# Nationwide population based epidemiological characteristics of complex regional pain syndrome in South Korea

**DOI:** 10.1038/s41598-026-45686-9

**Published:** 2026-04-13

**Authors:** Sohyeon Park, Seok-Ju Jeong, Joung Hwan Back, Minseok Oh, Chang-Gue Son, Eun-Jung Lee

**Affiliations:** 1 Department of Korean Rehabilitation Medicine, College of Korean Medicine, Daejeon University, 75, Daedeok-daero, 176 beongil, Seo-gu, Daejeon, 35235 Republic of Korea; 2 Health Insurance Review & Assessment Service, Dunsanbuk-ro 121, Seo- gu, Daejeon, 35236 Republic of Korea; 3 Convergence and Open Sharing System in Biohealth Sciences Project Group, Daejeon University, 62, Daehak-ro, Dong-gu, Daejeon, 34520 Republic of Korea; 4 Liver-Immunology Research Center, Daejeon University, 176 Daedeok- daero, Seo-gu, Daejeon, 35235 Republic of Korea

**Keywords:** Complex regional pain syndrome, epidemiology, prevalence, incidence, temporal trends, population-based study, Diseases, Health care, Medical research, Risk factors

## Abstract

Complex regional pain syndrome (CRPS) is a chronic and disabling pain disorder with limited nationwide epidemiological data. We conducted a population-based, repeated cross-sectional study using Korean Health Insurance Review and Assessment Service data from 2013 to 2022. CRPS cases were identified using Korean Classification of Diseases codes. Annual incidence and prevalence per 100,000 population were calculated and stratified by subtype, sex, and age. Crude rates showed a consistent decline in CRPS type I incidence and prevalence during the study period. Temporal trends were evaluated using negative binomial regression. Model 1 estimated overall temporal trends while adjusting for age and sex. CRPS type I demonstrated significant annual declines in incidence (20.5 to 6.5; IRR = 0.91, *p* < 0.001) and prevalence (30.6 to 16.8; PR = 0.93, *p* < 0.001), whereas type II showed no significant changes. Model 2 incorporated interaction terms between calendar year and demographic variables to assess subgroup differences. Although the overall decreasing trend for type I remained significant, age–year interactions were identified, with individuals aged ≥ 80 years demonstrating stable or modestly increasing patterns. These findings reveal subtype-specific temporal patterns and demographic heterogeneity in nationwide CRPS trends, offering updated epidemiological data to support future CRPS healthcare planning.

## Introduction

Complex regional pain syndrome (CRPS) is a chronic pain disorder characterized by atypical regional pain, burning sensations, hyperalgesia, and autonomic dysfunction^1^. It typically develops after tissue or nerve injury resulting from trauma, surgery, or immobilization^2^. The condition involves complex interplay of inflammation, nerve damage, and sympathetic nervous system dysfunction, making diagnosis and treatment particularly challenging^3,4^.

The International Association for the Study of Pain (IASP) classifies CRPS into two subtypes based on the presence or absence of confirmed nerve injury^5^. CRPS type I, formerly known as reflex sympathetic dystrophy (RSD), is defined by the absence of confirmed nerve injury and accounts for approximately 90% of cases^6^. CRPS type II, previously termed causalgia, involves verified nerve injury and is associated with more severe symptoms and a poorer prognosis^7^. These subtypes may differ in pathophysiology, treatment response, and epidemiological characteristics^2,8^.

Although multiple mechanisms - such as peripheral and central sensitization, neurogenic inflammation, sympathetic dysfunction, and genetic predisposition - have been proposed, the pathophysiology of CRPS remains poorly understood^3,9^. Due to an absence of objective biomarkers, diagnosis relies largely on clinical criteria, which has led to significant variability in reported incidence rates across populations and healthcare systems^10^. The Budapest criteria, revised by the IASP in 2003, improved diagnostic specificity but still depend heavily on clinical judgment^11,12^. Consequently, annual CRPS incidence has been reported to range widely, from 5.46 per 100,000 in the United States to 26.2 per 100,000 in the Netherlands^13,14^.

In South Korea, CRPS was formally incorporated into the Korean Standard Classification of Diseases (KCD) with the 5th revision implemented in 2008. Since then, the diagnostic coding for CRPS has undergone two major revisions, most recently in 2021, reflecting evolving clinical awareness and national health policy changes. Despite these developments, existing Korean studies have reported inconsistent epidemiological findings^15,16^. Two prior studies estimated national incidence rates ranging from 15.83 to 29.0 per 100,000 persons, but presented conflicting temporal trends for CRPS type I: one indicated an increasing trend, while the other suggested a slight decline.

These discrepancies highlight the need for updated, long-term epidemiological analysis that reflects the most recent diagnostic criteria. In this context, the South Korean healthcare system provides an ideal setting for nationwide research. The Health Insurance Review and Assessment Service (HIRA) database, which covers approximately 97% of the Korean population, contains comprehensive claims data that enable robust longitudinal analysis of disease incidence, prevalence, and demographic variation^17^.

Therefore, this study aimed to investigate national trends in the incidence and prevalence of CRPS and its subtypes over a 10-year period (2013–2022) using HIRA claims data. In addition, we examined interaction effects between age, sex, and temporal trends. This study seeks to provide an updated and comprehensive epidemiological understanding of CRPS within the Korean population.

## Methods

### Study design and population

This population-based, repeated cross-sectional study analyzed temporal trends in CRPS epidemiology in South Korea over a 10-year period (2013–2022). While the HIRA database was accessible from 2010, the data from 2010 to 2012 were intentionally utilized as a three-year wash-out period to ensure the accurate identification of incident cases. By excluding any individuals with a CRPS diagnosis during this preceding period, we ensured that cases identified from 2013 were strictly newly diagnosed (incident) rather than pre-existing prevalent cases. The study population comprised all individuals diagnosed with CRPS and treated through the Korean healthcare system during the study period.

### Data collection

Medical records of patients diagnosed with CRPS between January 1, 2013, and December 31, 2022, were obtained from the HIRA claims database.

Patients were identified using KCD codes corresponding to CRPS. The KCD codes included revisions during the study period: G5780, G5880, and G5881 were used from 2016 to 2019, and G905, G906, and G907 were used from 2020 (Table 1). M890 cases were included in the CRPS type I analysis for the entire study period, as algoneurodystrophy has traditionally been regarded as a variant of CRPS type I in clinical practice.

**Table 1:** Changes in CRPS diagnosis codes according to revisions in the KCD system.

KCD code	Revision version of KCD (Implementation year)			
	5th (2008 - 2010)	6th (2011 - 2015)	7th (2016 - 2020)	8th (2021 - present)
M890	CRPS type 1			Algoneurodystrophy
G905				CRPS type 1
G906				CRPS type 2
G564	CRPS type 2			
G5780			CRPS type 2 (Lower limb)	
G5880			CRPS type 2 (Other)	
G5881			CRPS type 2 (Multiple)	
G907				CRPS unspecified type (Other)

Data show the evolution of CRPS diagnostic codes across different revisions of the Korean Standard Classification of Diseases (KCD) system from 2008 to present. Shaded areas indicate periods when specific codes were in use. CRPS, Complex Regional Pain Syndrome; KCD, Korean Standard Classification of Diseases.

Data extracted included the annual number of patients with CRPS, the number of patients newly diagnosed with CRPS each year, and demographic characteristics including CRPS type, age, and sex. Patients were included only if they received treatment at hospitals, clinics, nursing hospitals, or Korean medical hospitals, while those visiting pharmacies or dental clinics were excluded. The study was approved for exemption from ethical review by the Institutional Bioethics Committee of Daejeon Oriental Medicine Hospital of Daejeon University (approval number: DJDSKH-21-E-23-1).

### Statistical analysis

The annual prevalence of CRPS was calculated by dividing the total number of patients diagnosed with CRPS by the mid-year population. Incidence was defined as the number of newly diagnosed CRPS cases per year divided by the total population. Both prevalence and incidence rates were stratified by CRPS subtype, sex, and age group. Age groups were categorized into 10-year intervals (< 20, 20–29, 30–39, 40–49, 50–59, 60–69, 70–79, 80–89 years). Population data were obtained from the Korean Statistical Information Service (KOSIS) provided by Statistics Korea.

Negative binomial regression models, also called as gamma-Poisson regression, were used to estimate the effect of calendar year on prevalence and incidence. Prevalence ratios (PR) and incidence rate ratios (IRR) were calculated to assess temporal changes in prevalence and incidence, respectively. Negative binomial regression was chosen over Poisson regression to account for potential overdispersion in the count data. Analyses were conducted separately for CRPS type I and type II and for each outcome measure. Model I assessed the overall temporal trend, adjusting for sex and age. Model II evaluated interaction effects between calendar year and demographic variables (sex and age) to identify subgroup-specific differences in time trends.

All statistical analyses were performed using STATA version 18.0 (StataCorp LLC, College Station, TX, USA), and a p-value < 0.05 was considered statistically significant.

## Results

### Incidence and prevalence of CRPS

During the 10-year study period (2013–2022), a total of 99,601 individuals were newly diagnosed with CRPS, corresponding to a mean annual incidence rate of 22.3 per 100,000 population (95% confidence interval (CI): 16.8–36.3). The mean annual incidence rates were 13.5 (95% CI: 10.6–24.1) for CRPS type I and 5.6 (95% CI: 5.1–10.7) per 100,000 for type II, yielding a type I to type II ratio of 2.4:1. Based on crude patient counts, the overall female-to-male ratio for incidence was 1.5:1 (Table 2).

**Table 2:** Number of newly diagnosed CRPS patients and incidence rate each year.

Year	Type 1					Type 2					Total			
	Male	Female	Total	F / M ratio	Incidence rate	Male	Female	Total	F / M ratio	Incidence rate	Total (NOS)	Type 1 / 2 ratio	F / M ratio	Incidence rate (NOS)
2013	3,927	6,458	10,385	1.6	20.5	1,415	1,931	3,346	1.4	6.6	13,731	3.1	1.6	27.2
2014	3,861	6,319	10,180	1.6	20.1	1,099	1,568	2,667	1.4	5.3	12,847	3.8	1.6	25.3
2015	3,477	5,449	8,926	1.6	17.5	986	1,261	2,247	1.3	4.4	11,173	4.0	1.5	21.9
2016	2,986	4,787	7,773	1.6	15.2	1,236	1,422	2,658	1.2	5.2	10,431	2.9	1.5	20.4
2017	2,550	4,164	6,714	1.6	13.1	1,411	1,760	3,171	1.2	6.2	9,885	2.1	1.5	19.3
2018	2,241	3,392	5,633	1.5	11.0	1,291	1,619	2,910	1.3	5.7	8,543	1.9	1.4	16.7
2019	2,111	3,184	5,295	1.5	10.3	1,278	1,783	3,061	1.4	6.0	8,356	1.7	1.5	16.3
2020	1,949	2,694	4,643	1.4	9.0	1,052	1,373	2,425	1.3	4.7	7,068	1.9	1.4	13.8
2021	2,647	3,338	5,985	1.3	11.7	1,412	1,529	2,941	1.1	5.7	10,003 (1,077)	2.0	1.2	19.5 (2.1)
2022	1,261	2,080	3,341	1.6	6.5	1,284	1,666	2,950	1.3	5.8	7,564 (1,273)	1.1	1.5	14.8 (2.5)
Total	40,259	64,060	68,875	1.6		17,750	22,897	28,376	1.3		99,601 (2,350)	2.4	1.5	
Mean (95% CI)	2,701	4,187	6,888		13.5 (10.6, 24.1)	1,246	1,591	2,838		5.6 (5.1, 10.7)	9,960			22.3 (16.8, 36.3)
SD	866	1,516	2,371		4.7	154	205	338		0.7	2184			6.6

Data represent annual numbers of newly diagnosed patients and age-standardized incidence rates (per 100,000 population) from Korean National Health Insurance Service claims data (2013–2022). F/M ratios represent overall ratios calculated from total patient numbers across the entire 10-year period. Total includes patients with non-otherwise specified CRPS diagnoses added since 2021. CRPS, Complex Regional Pain Syndrome; F/M ratio, female/male ratio; NOS, not otherwise specified; CI, confidence interval; SD, standard deviation.

Regarding prevalence, an annual average of 16,124 patients received CRPS-related treatment, with a mean annual prevalence of 31.6 per 100,000 (95% CI: 27.4–59.0). Type I patients outnumbered type II patients by a ratio of 3.1:1, and the crude female-to-male ratio for prevalence was 1.4:1 (Table 3).

**Table 3:** Number of treated CRPS patients and the prevalence rate each year.

Year	Type 1					Type 2					Total			
	Male	Female	Total	F / M ratio	Prevalence rate	Male	Female	Total	F / M ratio	Prevalence rate	Total (NOS)*	Type 1 / 2 ratio	F / M ratio	Prevalence Rate (NOS)
2013	5,834	9,615	15,449	1.6	30.6	2,527	2,937	5,464	1.2	10.8	20,913	2.8	1.5	41.4
2014	6,106	10,209	16,315	1.7	32.1	2,202	2,685	4,887	1.2	9.6	21,202	3.3	1.6	41.8
2015	5,965	9,486	15,451	1.6	30.3	1,975	2,046	4,021	1.0	7.9	19,472	3.8	1.5	38.2
2016	5,392	8,341	13,733	1.5	26.9	1,744	1,705	3,449	1.0	6.8	17,182	4.0	1.4	33.6
2017	4,792	7,430	12,222	1.6	23.9	1,798	1,861	3,659	1.0	7.1	15,881	3.3	1.4	31.0
2018	4,098	6,064	10,162	1.5	19.8	1,790	1,821	3,611	1.0	7.0	13,773	2.8	1.3	26.9
2019	4,049	5,953	10,002	1.5	19.5	1,765	1,865	3,630	1.1	7.1	13,632	2.8	1.3	26.6
2020	3,906	5,359	9,265	1.4	18.0	1,671	1,846	3,517	1.1	6.9	12,782	2.6	1.3	24.9
2021	3,860	5,261	9,121	1.4	17.8	1,412	1,529	2,941	1.1	5.7	13,139 (1,077)*	3.1	1.3	25.6 (2.1)
2022	3,582	5,052	8,634	1.4	16.8	1,459	1,662	3,121	1.1	6.1	13,268 (1,513)*	2.8	1.4	25.9 (3.0)
Total	47,584	72,770	120,354	1.5		18,343	19,957	38,300	1.1		161,244 (2,590)*	3.1	1.4	
Mean (95% CI)	4,758	7,277	12,035		23.6 (19.9, 43.4)	1,834	1,996	3,830		7.5 (6.5, 14.0)	16,124			31.6 (27.4, 59.0)
SD	983	2,000	2,980		6.0	333	455	780		1.6	3.4			4.8

Data represent annual patient numbers and age-standardized prevalence rates (per 100,000 population) from Korean National Health Insurance Service claims data (2013–2022). F/M ratios represent overall ratios calculated from total patient numbers across the entire 10-year period. Total includes patients with non-otherwise specified CRPS diagnoses added since 2021. CRPS, Complex Regional Pain Syndrome; F/M ratio, female/male ratio; NOS, not otherwise specified; CI, confidence interval; SD, standard deviation.

### Temporal trends in CRPS incidence and prevalence

During the study period, CRPS type I demonstrated a clear downward trend in both incidence and prevalence, whereas type II remained relatively stable.

#### Incidence

The incidence of type I decreased markedly from 20.5 per 100,000 in 2013 to 6.5 per 100,000 in 2022, corresponding to a 9% annual decline (IRR = 0.91, 95% CI: 0.88–0.95, *p* < 0.01). In contrast, type II incidence fluctuated between 4.4 and 6.6 per 100,000 but showed no statistically significant change over time (IRR = 0.99, 95% CI: 0.96–1.03, *p* = 0.58). Age-specific analyses indicated that both types were more common in older populations, with peak incidence observed in individuals in their 60s for type I and in their 70s for type II. After adjusting for age and year, females had a 36% higher incidence of type I (IRR = 1.36, 95% CI: 1.09–1.70, *p* = 0.01) and a 28% higher incidence of type II (IRR = 1.28, 95% CI: 1.06–1.56, *p* = 0.01) (Table 4).

**Table 4:** Trends in CRPS incidence by type.

	Type1					Type 2				
	IRR	SE	z	*P*-value	95% CI	IRR	SE	z	*P*-value	95% CI
***Model 1***										
Year	0.91	0.02	-4.58	<0.001*	0.88 - 0.95	0.99	0.02	-0.55	0.58	0.96 - 1.03
Age										
< 20(*Ref*)										
20-29	6.91	1.54	8.67	<0.001*	4.46 - 10.70	6.96	1.43	9.41	<0.001*	4.64 - 10.42
30-39	10.37	2.31	10.52	<0.001*	6.70 - 16.03	11.54	2.36	11.93	<0.001*	7.72 - 17.24
40-49	17.74	3.94	12.94	<0.001*	11.48 - 27.42	18.92	3.87	14.38	<0.001*	12.67 - 28.25
50-59	28.11	6.25	15.01	<0.001*	18.18 - 43.46	31.78	6.49	16.94	<0.001*	21.30 - 47.43
60-69	28.37	6.31	15.03	<0.001*	18.34 - 43.88	36.51	7.45	17.62	<0.001*	24.47 - 54.48
70-79	23.39	5.2	14.16	<0.001*	15.12 - 36.17	36.81	7.52	17.65	<0.001*	24.67 - 54.93
80-	4.33	0.78	8.17	<0.001*	3.05 - 6.16	8.72	1.51	12.48	<0.001*	6.21 - 12.25
Sex										
Male(*Ref*)										
Female	1.36	0.15	2.69	0.01*	1.09 - 1.70	1.28	0.13	2.5	0.01*	1.06 - 1.56
***Model 2***										
Age × Year										
< 20(*Ref*)										
20-29	1.07	0.08	0.87	0.39	0.92 - 1.24	1.09	0.05	1.78	0.08	0.99 - 1.20
30-39	1.04	0.08	0.46	0.64	0.89 - 1.20	1.08	0.05	1.74	0.08	0.99 - 1.17
40-49	1.02	0.08	0.29	0.77	0.88 - 1.19	1.07	0.04	1.68	0.09	0.99 - 1.16
50-59	1.02	0.08	0.29	0.78	0.88 - 1.18	1.06	0.04	1.45	0.15	0.98 - 1.15
60-69	1.07	0.08	0.87	0.38	0.92 - 1.24	1.1	0.04	2.27	0.02*	1.01 - 1.19
70-79	1.05	0.08	0.58	0.56	0.90 - 1.21	1.06	0.05	1.45	0.15	0.97 - 1.15
80-	1.13	0.07	1.95	0.05	1.00 - 1.27	1.19	0.45	2.66	0.01*	1.05 - 1.35
Sex × Year										
Male(*Ref*)										
Female	0.99	0.03	-0.39	0.7	0.93 - 1.05	1.02	0.03	0.75	0.45	0.960 - 1.08

Data represent incidence rate ratios (IRR) with 95% confidence intervals (CI) from negative binomial regression analysis of Korean National Health Insurance Service claims data (2013-2022). Model 1: individual effects without time interaction; Model 2: including time interaction effects. Reference categories: age <20 years, male sex. CRPS, Complex Regional Pain Syndrome; SE, standard error; Z, z-score. *p < 0.05.

#### Prevalence

The prevalence of type I declined from 30.6 per 100,000 in 2013 to 16.8 per 100,000 in 2022, corresponding to a 7% annual decrease (PR = 0.93, 95% CI: 0.89–0.97, *p* < 0.01). Type II prevalence declined from 10.8 per 100,000 to 6.1 per 100,000 during the same period, but the trend did not reach statistical significance (PR = 0.97, 95% CI: 0.93–1.00, *p* = 0.08). Similar to incidence, prevalence rates were higher in older age groups, with peak prevalence in the 60s for type I and the 70s for type II. After adjustment, females had a significantly higher prevalence of type I (PR = 1.36, 95% CI: 1.07–1.74, *p* = 0.01), whereas no significant sex difference was found for type II (PR = 1.08, 95% CI: 0.87–1.35, *p* = 0.47) (Table 5).

**Table 5:** Trends in CRPS prevalence by type.

	Type1					Type 2				
	PR	SE	z	*P*-value	95% CI	PR	SE	z	*P*-value	95% CI
**Model 1**										
Year	0.93	0.02	-3.32	<0.001*	0.89 - 0.97	0.97	0.02	-1.76	0.08	0.93 - 1.00
Age										
< 20(*Ref*)										
20-29	8.98	2.19	9	<0.001*	5.57 - 14.48	8.49	1.96	9.29	<0.001*	5.41 - 13.33
30-39	13.06	3.18	10.57	<0.001*	8.11 - 21.04	15.78	3.62	12.04	<0.001*	10.07 - 24.73
40-49	22.22	5.39	12.77	<0.001*	13.80 - 35.76	26.56	6.07	14.34	<0.001*	16.97 - 41.58
50-59	36.58	8.88	14.82	<0.001*	22.72 - 58.88	44.48	10.16	16.62	<0.001*	28.43 - 69.59
60-69	41.54	10.1	15.32	<0.001*	25.79 - 66.91	53.76	12.28	17.45	<0.001*	34.36 - 84.10
70-79	37.47	9.12	14.89	<0.001*	23.26 - 60.36	55.96	12.78	17.62	<0.001*	35.77 - 87.55
80-	6.09	1.18	9.32	<0.001*	4.17 - 8.91	12.18	2.3	13.22	<0.001*	8.41 - 17.64
Sex										
Male(*Ref*)										
Female	1.36	0.17	2.49	0.01*	1.07 - 1.74	1.08	0.12	0.72	0.47	0.87 - 1.35
**Model 2**										
Age × Year										
< 20(*Ref*)										
20-29	1.07	0.09	0.77	0.44	0.90 - 1.26	1.07	0.05	1.34	0.18	0.97 - 1.18
30-39	1.06	0.09	0.7	0.48	0.90 - 1.25	1.07	0.05	1.6	0.11	0.98 - 1.17
40-49	1.04	0.09	0.46	0.65	0.88 - 1.23	1.06	0.04	1.5	0.13	0.98 - 1.15
50-59	1.03	0.09	0.35	0.73	0.87 - 1.21	1.04	0.04	0.89	0.37	0.96 - 1.12
60-69	1.07	0.09	0.87	0.39	0.91 - 1.27	1.06	0.04	1.55	0.12	0.98 - 1.15
70-79	1.06	0.09	0.7	0.49	0.90 - 1.25	1.03	0.04	0.66	0.51	0.95 - 1.12
80-	1.16	0.08	2.2	0.03*	1.02 - 1.32	1.14	0.08	1.91	0.06	1.00 - 1.30
Sex × Year										
Male(*Ref*)										
Female	0.98	0.03	-0.63	0.53	0.92 - 1.05	1.01	0.03	0.46	0.64	0.95 - 1.08

Data represent prevalence ratios (PR) with 95% confidence intervals (CI) from negative binomial regression analysis of Korean National Health Insurance Service claims data (2013-2022). Model 1: individual effects without time interaction; Model 2: including time interaction effects. Reference categories: age < 20 years, male sex. CRPS, Complex Regional Pain Syndrome; SE, standard error; Z, z-score. *p < 0.05.

#### Interaction effects

**Fig. 1:**
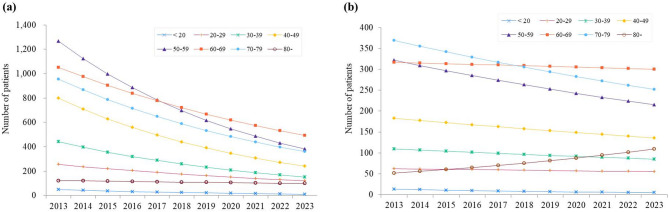
Predicted incidence trends by age group and CRPS type. (**a**) CRPS Type I (**b**) CRPS Type II. Different colored lines represent different age groups (10-year intervals from < 20 to ≥ 80 years). Lines show temporal trends from negative binomial regression models.

**Fig. 2:**
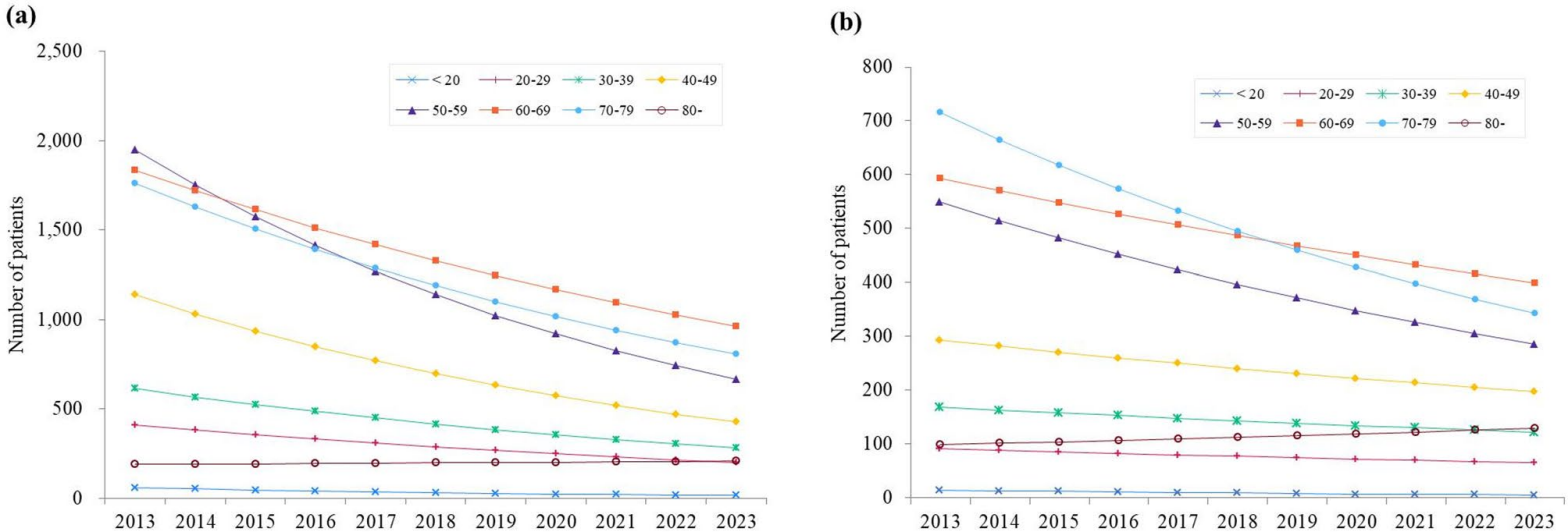
Predicted prevalence trends by age group and CRPS type. (**a**) CRPS Type I (**b**) CRPS Type II. Different colored lines represent different age groups (10-year intervals from < 20 to ≥ 80 years). Lines show temporal trends from negative binomial regression models.

Interaction analyses using Model 2 confirmed that the overall downward temporal trends for CRPS type I remained robust across most age groups after adjusting for age and sex. However, significant age-specific interactions were identified, particularly in the oldest age group. For type I, individuals aged ≥ 80 years showed a modestly increased incidence (IRR = 1.13, 95% CI: 1.00–1.27, *p* = 0.05) and a significantly higher prevalence (PR = 1.16, 95% CI: 1.02–1.32, *p* = 0.03) compared with younger groups (Tables 4 and 5; Figs. 1a and 2a). For type II, individuals aged ≥ 80 years demonstrated a marked increase in incidence (IRR = 1.19, 95% CI: 1.05–1.35, *p* = 0.01) and those in their 60s also showed a significant but smaller increase (IRR = 1.10, 95% CI: 1.01–1.19, *p* = 0.02). In contrast, type II prevalence among individuals aged ≥ 80 years remained relatively stable, with only a slight, non-significant upward trend (PR = 1.14, 95% CI: 1.00–1.30, *p* = 0.06) (Tables 4 and 5; Figs. 1b and 2b). No significant sex–year interactions were identified for either incidence or prevalence (Tables 4 and 5).

## Discussion

Understanding the epidemiology and temporal trends of CRPS has important clinical and public health implications^18^. Such studies help optimize healthcare resource allocation, evaluate the effectiveness of diagnostic and therapeutic strategies, identify high-risk populations for targeted prevention, and provide insights into disease mechanisms^19^. In this context, this nationwide population-based study represents the most comprehensive epidemiological analysis of CRPS in Korea to date, covering a 10-year period (2013–2022) and analyzing both CRPS subtypes with demographic stratification. Our findings reveal distinct temporal patterns between CRPS subtypes that provide important insights for healthcare planning and patient management.

A key finding was the significant decline in both incidence and prevalence of CRPS type I, with average annual reductions of 9% and 7%, respectively (Tables 4 and 5). Interestingly, the strength of these associations showed a slight attenuation in Model 2 after adjusting for age and sex. This suggest that the observed decline is partly intertwined with South Korea’s shifting demographic structure, such as population aging, which modifies the pure effect of calendar time. Despite these demographic complexities, the overall declining trend remains clear and may result from several factors: broader implementation of the Budapest diagnostic criteria promoting more conservative diagnoses^11^, improvements in perioperative and trauma care, and increased utilization of evidence-based pain management strategies including early mobilization and multidisciplinary interventions^20,21^. Additionally, administrative coding changes significantly influenced these patterns. The separation of M890 (algoneurodystrophy) from CRPS coding in KCD-8 and its exclusion from catastrophic disease benefits likely discouraged less stringent diagnostic practices, as evidenced by the 35% decrease in M890 diagnoses between 2020 and 2022 (data not shown). Given that this study was based on nationwide administrative claims data, the observed temporal patterns should be interpreted as changes in diagnosed and treated cases within the healthcare system and may not fully represent underlying biological incidence.

In contrast, CRPS type II showed stable epidemiology throughout the study period (Figs. 1 and 2). This stability likely reflects type II’s reliance on objective evidence of nerve injury, making it less susceptible to diagnostic variations or administrative changes compared to type I, which lacks objective diagnostic markers^6^. The persistence of type II burden suggests that while current approaches effectively address type I, novel strategies are needed for type II prevention and management, including standardized protocols for patients at risk of nerve injury and specialized treatment programs for established cases^22,23^.

The observed demographic patterns align with international studies but provide novel insights for targeted interventions^24–27^. Female predominance (57.5% overall) was particularly pronounced in type I (1.5:1 ratio) compared to type II (1.1:1 ratio), consistent with previous research^24–26,28,29^(Table 3). This differential pattern suggests that sex-related factors may play different roles in the two CRPS subtypes^25,30^. Peak incidence in the 60–79 years age groups reflects higher rates of fractures, surgeries, and age-related comorbidities in this population^13,14^. Notably, patients ≥ 80 years showed stable or slightly increasing trends, possibly reflecting multimorbidity, reduced physiological reserve, and different healthcare utilization patterns in elderly populations^31^. This age-specific variation indicates that prevention and treatment strategies should be tailored according to age groups, with particular attention to elderly patients who may have different risk profiles and treatment responses.

Our study leverages the comprehensive HIRA database covering approximately 98% of the Korean population^17^, enabling robust longitudinal analysis. This study utilized 10 years of recent data (2013–2022) and incorporated the updated KCD-8 coding system, integrated analyses of both incidence and prevalence, and subtype-specific trends by age and sex to clarify the inconsistencies in CRPS incidence and trend reported in the 2018 and 2021 studies^15,16^. By extending previous Korean research, our findings provide a more comprehensive and refined understanding of the epidemiological patterns of CRPS.

However, several limitations must be acknowledged. First, administrative coding changes, particularly the redefinition of M890, complicate trend interpretation despite our consistent inclusion approach. Furthermore, evolving diagnostic practices and coding policies within the healthcare system may have influenced the number of recorded cases over time. Second, the claims-based methodology lacks clinical severity measures, treatment response data, and patient-reported outcomes that would enhance understanding of disease burden and therapeutic effectiveness. Third, we could not account for potential confounders such as changes in trauma rates, surgical volumes, or healthcare utilization patterns that might independently influence CRPS incidence.

Future research should clarify the mechanisms underlying subtype-specific trends, identify key interventions driving type I improvements, and develop targeted strategies for type II management through prospective studies incorporating clinical severity measures and long-term follow-up. Additionally, age-specific investigations, particularly in vulnerable populations such as elderly patients, are needed to enhance understanding of CRPS epidemiology and inform tailored management strategies.

## Conclusion

In conclusion, this 10-year nationwide study demonstrates a significant decline in CRPS type I incidence and prevalence, while type II rates remain stable. These divergent trends, alongside the increasing burden in the oldest age groups, reflect the complex interplay between evolving diagnostic practices and demographic aging. These findings provide a critical epidemiological benchmark for evaluating the long-term impact of diagnostic standardization and serve as a data-driven foundation for future healthcare resource allocation and the development of clinical management guidelines for CRPS.

## Data Availability

All data supporting the findings of this article are available from the corresponding author (E-J Lee).
